# Fulminant fatal pneumonia and bacteremia due to *Aeromonas dhakensis* in an immunocompetent man: a case report and literature review

**DOI:** 10.3389/fcimb.2024.1359422

**Published:** 2024-07-15

**Authors:** Lei Jiang, Qiquan Zhao, Dairong Li, Jia Gao, Xiaobing Zhang, Qian Shu, Xiaoli Han

**Affiliations:** ^1^ Department of Pulmonary and Critical Care Medicine, The First Affiliated Hospital of Chongqing Medical University, Chongqing, China; ^2^ Department of Pulmonary and Critical Care Medicine, The People’s Hospital of Dazu, Chongqing, China; ^3^ Department of Radiology, The First Affiliated Hospital of Chongqing Medical University, Chongqing, China; ^4^ Department of Clinical Laboratory, The First Affiliated Hospital of Chongqing Medical University, Chongqing, China; ^5^ Department of Pathology, The First Affiliated Hospital of Chongqing Medical University, Chongqing, China

**Keywords:** *Aeromonas dhakensis*, pneumonia, bacteremia, metagenomic next-generation sequencing, housekeeping gene sequencing

## Abstract

**Background:**

*Aeromonas dhakensis* is associated with soft tissue infection, bacteremia and gastroenteritis. Involvement of respiratory system in adults is extremely rare. We report a case of fulminant pneumonia and bacteremia due to *A. dhakensis* in a patient without underlying diseases.

**Case presentation:**

A 26-year-old man became ill suddenly with pneumonia after swimming in a river. Despite intensive support measures in the intensive care unit, he died 13 hours after admission and 4 days after his first symptoms. Autopsy showed abundant Gram-negative bacteria, massive inflammatory cell infiltration, edema, necrosis and hemorrhage in lung tissue. *A. dhakensis* was isolated from blood culture taken at admission and bronchoalveolar lavage fluid (BALF) after intubation. Moreover, *A. dhakensis* was also detected in lung tissue by metagenomic next-generation sequencing (mNGS) assay. The infection may have come from river water.

**Conclusion:**

In patients who develop a fulminant pneumonia after contacting an aquatic environment, *A. dhakensis* should be alerted and mNGS may aid in the detection of aquatic pathogens by being more sensitive and specific versus traditional bacterial culture.

## Introduction

*Aeromonas dhakensis*, is a facultative anaerobic Gram-negative bacillus and often isolated from aquatic environments, including rivers and lakes. It is emerging as an important human pathogen that can cause severe soft tissue infections, gastroenteritis, and fatal bloodstream infections (Shin et al., 2013; Chen et al., 2014b, 2016; Chang et al., 2018; Huang et al., 2020a). *A. dhakensis* (Huys et al., 2002) Beaz-Hidalgo et al., 2015, was first isolated from children with diarrhea and described as *A. hydrophila* subsp. *dhakensis* Huys et al., 2002 (Huys et al., 2002), and latter was also classified as *A. aquariorum* Martinez-Murcia et al., 2008 (Martinez-Murcia et al., 2008). After *A. hydrophila* subsp. *dhakensis* and *A. aquariorum* being confirmed as the same taxon, both of them have been reclassified as *A. dhakensis* sp. nov. comb nov (Beaz-Hidalgo et al., 2013, 2015). *A. dhakensis* bacteremia was observed to cause a higher mortality rate than non-*A. dhakensis* species (Chen et al., 2014b). A fatal case of *A. dhakensis* severe sepsis in an old man with liver cirrhosis was reported in Korea (Shin et al., 2013), two severe dengue patients with *A. dhakensis* bacteremia and necrotizing fasciitis passed out due to shock and multiorgan failure in southern Taiwan (Chang et al., 2018), and a young man with *A. dhakensis* septicemia accompanying chronic hepatitis B virus infection after the ingestion of a meal of raw snakehead fish died from multiple organ failure in Mainland China (Huang et al., 2020a). However, community-acquired bacterial pneumonia due to this organism is extremely rare, especially in a non-immunocompromised host. Here, we describe fulminant fatal pulmonary infection and bacteremia due to *A. dhakensis* in an immunocompetent man.

## Case report

A 26-year-old man was admitted to our hospital complaining of cough, fatigue, fever, hemoptysis and dyspnea. Three days before admission, the patient became ill after swimming in a river, his condition did not improve significantly after treatment with oral cefuroxime (0.25 g, every 12h) prescribed by a local clinic. One day prior to admission, the patient’s symptoms became exacerbated and included fever, sputum expectoration, chest pain, wheezing, shortness of breath and hemoptysis. He had no gastrointestinal complaints in his course of illness. He had a five-year history of light beer consumption. He had smoked approximately 20 cigarettes per day for 4 years. He had been in good health without history of diabetes mellitus, liver cirrhosis, chronic lung disease, immunosuppression, recurrent infection, malignancies, chemotherapy, or steroid use. His vital signs were as follows: respiratory rate, 18 breaths/minute; pulse rate, 82 beats/minute and regular; blood pressure, 129/81 mmHg; and temperature, 37.5°C. His heart sound was normal. Coarse breath sounds and scattered wet rales could be auscultated in the bilateral lower lungs.

**Figure 1:**
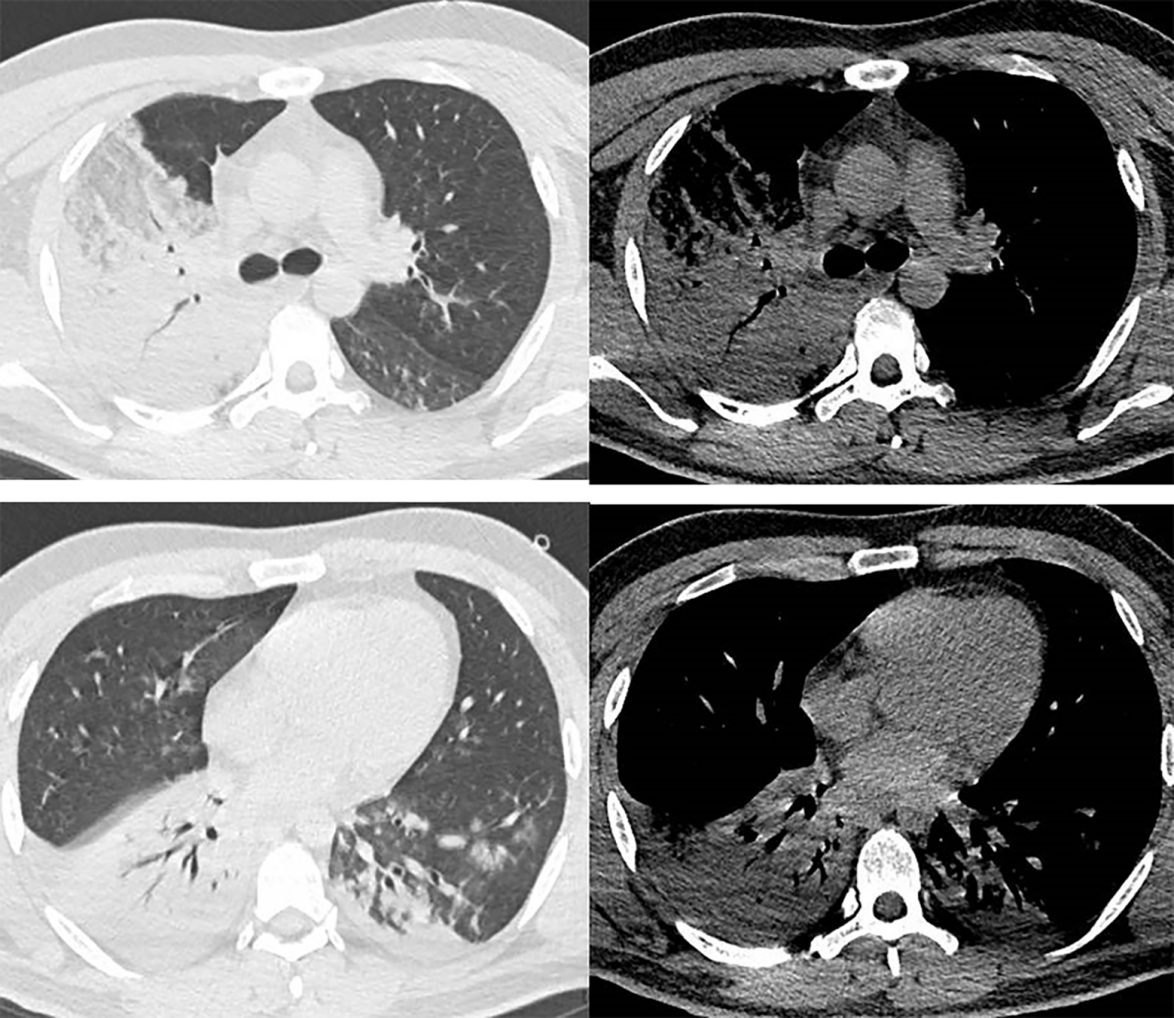
Images of chest computed tomography.

Laboratory findings on admission were as follows: white blood cell (WBC) 7060/mm^3^ (normal value 3500 - 9500/mm^3^); lymphocyte 1870/mm^3^ (normal value 1100 - 3200/mm^3^); alanine aminotransferase (ALT) 150 IU/L (normal value 13 - 69 IU/L); aspartate transferase (AST) 84 IU/L (normal value 15 - 46 IU/L); serum creatinine (Cre) 2.23 mg/dL (normal range: 0.66 -1.24 mg/dL); serum urea nitrogen (BUN) was 97.29 mg/dL (normal value 36.20 - 80.32 mg/dL); serum uric acid (UA) 6.87 mg/dL (normal value 2.35 - 5.72 mg/dL); C-reactive protein (CRP) was 8.67 mg/dL (normal value 0 - 0.8 mg/dL); Procalcitonin (PCT) 99.57 ng/mL (normal value 0 - 0.05 ng/mL); Prothrombin time international normalized ratio (PT-INR) was 1.87 (normal value 0 80 - 1.2); and D-dimer was 10.48 μg/mL (normal value 0 - 0.55 μg/mL). HIV antibody and rheumatological work-up were negative. Hepatitis B virus, hepatitis C virus infection and live cirrhosis were not detected. Arterial blood gas analysis revealed pH 7.32, PaCO_2_ 43 mmHg, PaO_2_ 37 mmHg, HCO_3_^-^ 22.21 mmol/L, Lac 4.8 mmol/L, SaO_2_ 65% with the patient breathing room air. Chest computed tomography(CT)scan on arrival at our hospital revealed consolidation in his right lung and an infiltrating shadow in his left lower lobe (**Figure 1**).

Non-invasive ventilation was begun and empirical antimicrobial treatment with imipenem-cilastatin (0.5 g: 0.5 g, every 8 h) and linezolid(0.6 g, every 12h)was intravenously administered 1 hour after his arrival. Two sets of blood cultures were obtained at the same time. However, his arterial blood gas tensions deteriorated rapidly. 3 hours later, he was intubated for invasive ventilation. Massive bloody secretions were suctioned from the endotracheal tube. Bronchoscopy demonstrated diffuse alveolar hemorrhage (DAH) and bronchoalveolar lavage fluid (BALF) was obtained for pathogen culture. The patient went into severe acute respiratory distress syndrome (ARDS), DAH and shock quickly. An extracorporeal membrane oxygenation (ECMO) evaluation was requested for severe ARDS and fast-deteriorating hypoxemia, and, given the likely reversible nature of his pulmonary disease and hypoxemia. However, his family refused to initiate ECMO support due to huge expense. Despite intensive supportive efforts, he eventually died 13 hours after admission.

**Figure 2:**
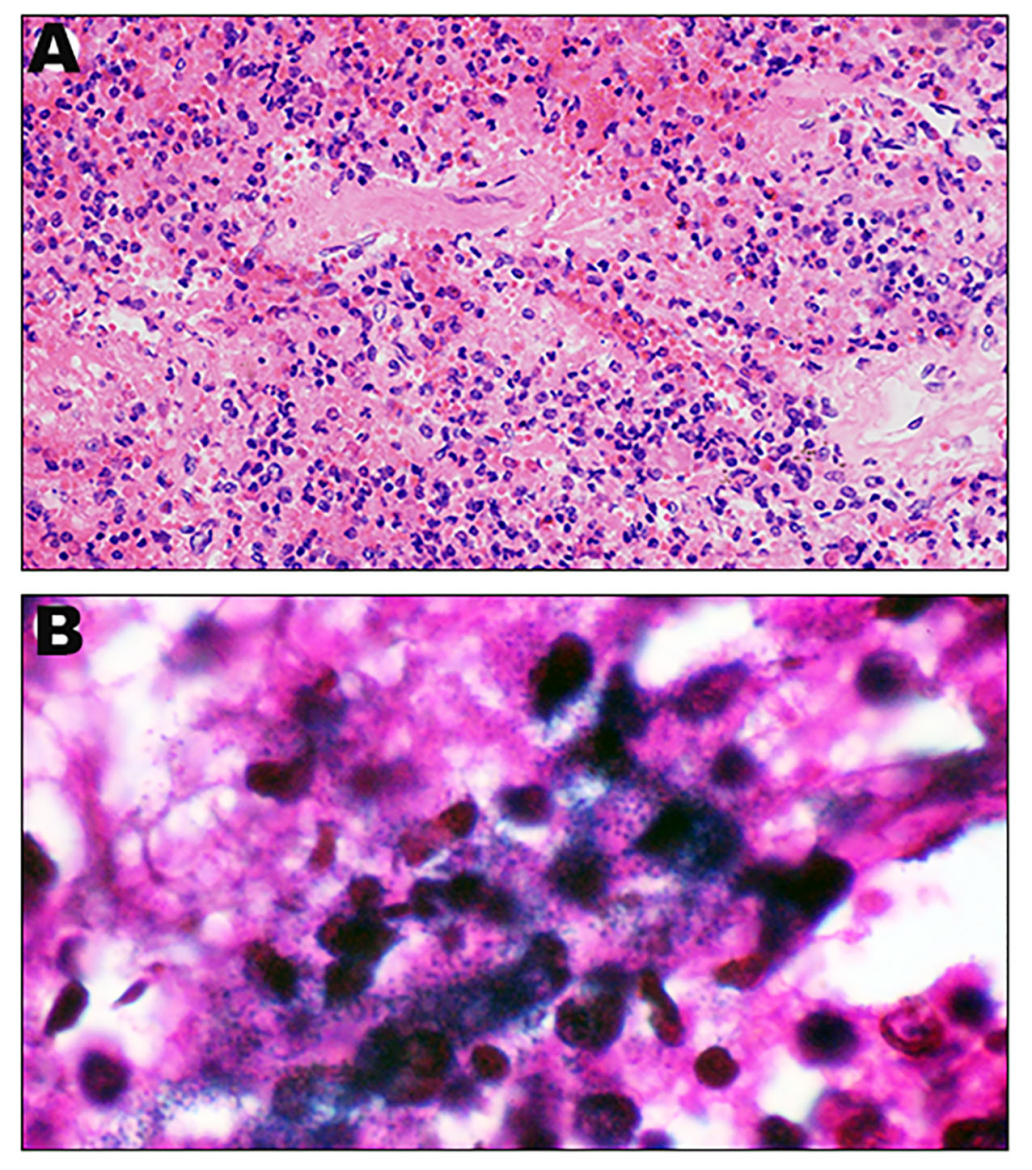
Histopathological appearance of lung: Representative H&E sections of lung (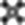
400) **(A)**. Representative Gram stain sections of lung (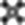
1000) **(B)**.

**Figure 3:**
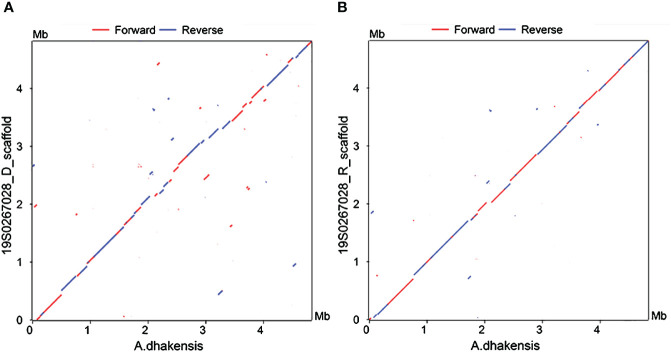
Nucleotide-based alignments with NUCmer. X-axis: reference genome of *A dhakensis*. Y-axis: **(A)** 19S0267028_D and **(B)** 19S0267028_R. Aligned segments are presented as dots or lines in the NUCmer alignment and were generated by the MUMmer plot script.

We sought to further confirm the diagnosis after death, a percutaneous lung biopsy was performed to obtain lung tissue samples for pathological examination and metagenome next-generation sequencing (mNGS) assay performed on a BGISEQ-500 platform (Beijing Genomics Institute, Wuhan, China). Microscopically lung tissue was infiltrated with leucocytes and the alveoli were flooded with fluid and erythrocytes. Zones of edema, necrosis and hemorrhage were present (**Figure 2A**). Numerous small Gram-negative bacteria were seen with inflammatory cells (**Figure 2B**). The BALF and two sets of blood cultures all yielded *Aeromonas hydrophila*, identified by VITEK^®^ MS MALDI-TOF system (BioMeírieux, Marcy I’ Etoile, France) but re-identified as *A*. *dhakensis* (accession number: GCF_905132925.1) by housekeeping gene sequencing (*rpoD* and *gyrB*) as described in the previous study (Zhang et al., 2023). mNGS of the lung puncture tissue also suggested *A. dhakensis*. A total of 100226 unique DNA reads and 103244 RNA reads mapping of the *A. dhakensis* genome were reported. The raw mNGS sequence results have been uploaded to the NCBI data (accession number: PRJNA1120325). To confirm the strain’s identification as *A. dhakensis*, the sequences were aligned with the reference genome of *A. dhakensis* (accession number: GCF_905132925.1), as detailed in **Supplementary Material 1**. The MUMmer analysis showed a red line in the forward direction and a blue line in the reverse direction, indicating that the genomes were mostly co-linear (**Figure 3**). A substantial similarity of 80% between the two was noted. The test for *A. dhakensis* susceptibility to various antimicrobial agents was performed by the microbroth dilution method according to Clinical and Laboratory Standards Institute (CLSI) 2015 guidelines (CLSI, 2015), which revealed that *A. dhakensis* was resistant to trimethoprim-sulfamethoxazole and cefoxitin, and sensitive to piperacillin tazobactam, imipenem, meropenem, levofloxacin, ciprofloxacin, cefuroxime, cefepime, ceftriaxone, cefoperazone sulbactam, ceftazidime and amikacin.

## Discussion and conclusions

This is a rare report of fatal hemorrhagic pneumonia and bacteremia due to *A. dhakensis* in a fit young man. Spontaneous bacterial peritonitis, soft-tissue infection, biliary tract infection and primary bacteremia are major origins of *A. dhakensis* bacteremia (Kitagawa et al., 2020). Pneumonia as a source of *A. dhakensis* bacteremia has been mentioned in an earlier study (Wu et al., 2015). It also has been reported that *A. dhakensis* can be recovered from the respiratory tract or lung with a low rate (Fernandez-Bravo and Figueras, 2020; Puah et al., 2022). Here, pneumonia may well be the source of *A. dhakensis* bacteremia, because it was found that massive bacteria were scattered in lung tissue and isolated from both bronchial secretion and blood in this case.

*Aeromonas* bacteremia usually occurs in patients with weakened immune system and underlying conditions including liver cirrhosis, diabetes mellitus, malignancy etc (Wu et al., 2015). However, no predisposing underlying diseases were identified in this patient. Despite immediate and intensive therapy with antibiotics, the patient’s medical condition acutely deteriorated and the patient died. Fulminant pneumonia due to *A. dhakensis* after swimming showed a too rapid fatal clinical course for the therapy to be effective. Due to the unknown etiology, infection severity, sepsis and rapid clinical decline, a broad-spectrum antibiotic (imipenem) and an antibiotic with methicillin resistant staph aureus coverage were used in our patient initially. However, volume of distribution (V_d_) of imipenem could change according to the severity of illness, inflammation, and shock. The high V_d_ of imipenem may lead to subtherapeutic concentrations among sepsis or septic shock patients and further influence their prognosis (Huang et al., 2020c). A high loading dose of imipenem should be considered in the treatment of sepsis or septic shock (Huang et al., 2020b). In addition, a high prevalence of carbapenem resistance in *A. dhakensis* clinical isolates has been reported (Puah et al., 2022). Monotherapy with a routine dose of imipenem may not be an appropriate treatment choice for this case with high *A. dhakensis* burden in lung tissue and blood. However, the antimicrobial susceptibility tests performed on the *A. dhakensis* strain after the patient’s death showed susceptibility to both imipenem and meropenem. *Aeromonas* spp. exhibited best susceptibility to aminoglycosides (Sun et al., 2021), suggesting that aminoglycosides might be recommended for initial therapy of *Aeromonas*-associated bacteremia. Therefore, a higher dose of imipenem plus aminoglycosides might be required in *A. dhakensis* pneumonia and bacteremia empirically.

ECMO can provide temporary pulmonary assistance to prolong the time frame for diagnosis and specific treatment in severe ARDS patients (Resch et al., 2009). It has also been reported that ECMO was feasible in ARDS along with DAH (Abrams et al., 2015; Zhang and Yu, 2022). VV-ECMO as a bridging strategy to pulmonary recovery was successfully used in a trauma patient with fulminant *A. hydrophila* pneumonia and ARDS (Issa and Napolitano, 2011). Thus, ECMO may be effective to provide oxygenation support, ensure sufficient time to initiate treatment measures and allow for further therapy to take effect in this patient. When invasive mechanical ventilation fails to maintain adequate blood oxygen levels, ECMO should be initiated immediately, especially in the patients with reversible and fulminant severe pneumonia. Meanwhile, hemorrhagic complications and aggravated DAH should be avoided by low-level, delayed, or even no systemic anticoagulation.

The majority of *Aeromonas* pneumonia cases has been reported after freshwater or saltwater near drowning (Reines and Cook, 1981; Goncalves et al., 1992; Ender et al., 1996; Ender and Dolan, 1997; Miyake et al., 2000; Cousin and Pittet, 2024). *A. hydrophila* was the most common aeromonad reported to cause pneumonia in drowning patients previously (Ender and Dolan, 1997). *Aeromonas* pneumonia is possibly due to the aspiration of a large amount of water containing a high concentration of bacteria that can quickly lead to hemorrhagic pneumonia. Therefore, the most important relevant factor in this case may be his recent swim in the river. In the environment, *A. dhakensis* has been recovered from river water, cooling-system water pond, fish tank water and fish (Chen et al., 2016; Huang et al., 2020a). Oral ingestion or abraded open wounds can serve as the portals of entry of *A. dhakensis* infection (Janda and Abbott, 2010). In immunocompetent hosts, respiratory infection due to this pathogen may occur by ingestion or even aspiration of contaminated water as documented in previous studies (Miyake et al., 2000; Mukhopadhyay et al., 2003). In addition, predisposing factors for *Aeromonas* pneumonia involve alcohol and cigarette consumption (Baddour and Baselski, 1988) and our patient consumed alcohol regularly and smoked 20 cigarettes per day. His bad habits may leave him vulnerable to *A. dhakensis* infection.

According to the literature, *A. dhakensis* bacteremia is more lethal than bacteremia due to other *Aeromonas* species (Wu et al., 2015; Chen et al., 2016; Zhang et al., 2023). Moreover, *A. dhakensis* is more virulent than *A. hydrophila* and an independent mortality risk factor in monomicrobial *Aeromonas* bacteremia (Wu et al., 2015). In the present study, the patient died from respiratory failure and septic shock 13 hours postadmission despite all intensive supportive efforts. The clinical features were hemoptysis, sepsis, progressive respiratory failure and high mortality rate. Histopathological examination showed severe hemorrhagic pneumonia with necrosis and numerous small gram-negative bacteria with a remarkable inflammatory cellular reaction, which may suggest the virulence of this pathogen and could explain the rapid clinical course. The pathogenesis of such an extremely rapid clinical course remains to be further clarified.

Pneumonia with *A. dhakensis* might be underreported, underdiagnosed, or misdiagnosed. Many similarities in the clinical features, predisposing factors and pathological manifestations exist between *A. dhakensis* pneumonia and *A. hydrophila* pneumonia. *A. dhakensis* is often misidentified as *A. hydrophila, A. veronii, or A. caviae* by commercial phenotypic tests in the clinical settings (Wu et al., 2015), which may bring about misdiagnosis and non-effective treatment. Corrective identification of the pathogen may be of great importance for the successful treatment of the patients with *A. dhakensis* infection, which relies on molecular identification with the sequences of housekeeping genes (Soler et al., 2004). In this patient, *A. dhakensis* in the blood was misidentified as *A. hydrophila, or A. caviae* by VITEK MALDI-TOF system, while it was identified by mNGS assay or *rpoD* sequencing correctly. Therefore, laboratories should be alerted to the possibility that *A. dhakensis* could be a potentially pathogenic bacterium with important antimicrobial resistances (Chen et al., 2014a, 2016; Puah et al., 2022). It has been reported that *A. dhakensis* could be accurately and efficiently identified by MALDI-TOF MS (Chen et al., 2014a; Fernandez-Bravo and Figueras, 2020). However, *A. dhakensis* is not included in the commercial database of MALDI-TOF system. Once *A. dhakensis* is added to the commercial MALDI-TOF database, it may be not difficult to accurately identify it (Fernandez-Bravo and Figueras, 2020; Kitagawa et al., 2020). Meanwhile, mNGS is also an effective tool for the detection of *A. dhakensis.*

In summary, although *A. dhakensis* rarely causes pneumonia, it does occur as a fulminant type of pneumonia occasionally in patients with or without underlying conditions. Clinicians should be rapidly aware of and accurately identify this rare organism, and select proper antibiotics with adequate doses in the treatment of *A. dhakensis* pneumonia and bacteremia. ECMO may be required in the rescue treatment of fulminant fatal pneumonia with DAH.

## Data availability statement

The data presented in the study are deposited in the NCBI repository, accession number PRJNA1120325.

## Ethics statement

The studies involving humans were approved by the Ethical Committee of the First Affiliated Hospital of Chongqing Medical Hospital. The studies were conducted in accordance with the local legislation and institutional requirements. The participants provided their written informed consent to participate in this study. Written informed consent was obtained from the individual(s) for the publication of any potentially identifiable images or data included in this article. Written informed consent was obtained from the participant/patient(s) for the publication of this case report.

## Author contributions

LJ: Conceptualization, Data curation, Funding acquisition, Writing – original draft. QZ: Data curation, Writing – review & editing. DL: Data curation, Methodology, Writing – review & editing. JG: Data curation, Writing – review & editing. XZ: Data curation, Methodology, Writing – review & editing. QS: Methodology, Writing – review & editing. XH: Conceptualization, Resources, Supervision, Writing – review & editing.
